# Employment is maintained and sick days decreased in psoriasis/psoriatic arthritis patients with etanercept treatment

**DOI:** 10.1186/1471-5945-14-14

**Published:** 2014-08-05

**Authors:** Robert L Boggs, Sarolta Kárpáti, Wenzhi Li, Theresa Williams, Ronald Pedersen, Lotus Mallbris, Robert Gniadecki

**Affiliations:** 1 Formerly of Pfizer Inc., 3921 Glenlake Garden Drive, Raleigh, NC 27612, USA; 2 Semmelweis University, Budapest, Hungary; 3 Pfizer Inc, Collegeville, PA, USA; 4 University of Copenhagen, Copenhagen, Denmark

**Keywords:** Psoriasis, Psoriatic arthritis, Pharmacoeconomics, Etanercept, Employment, Sick days

## Abstract

**Background:**

Psoriasis and psoriatic arthritis (PsA) impair quality of life, including reduction in employment or job duties. The PRESTA (Psoriasis Randomized Etanercept STudy in Patients with Psoriatic Arthritis) study, a randomized, double-blind, two-dose trial, examined the efficacy of etanercept treatment in patients with moderate-to-severe plaque psoriasis and PsA and the main results have been presented previously. This analysis examined employment status, job duties and sick days, pre-defined endpoints in PRESTA, among this patient population.

**Methods:**

Participants (N = 752) were randomized to receive etanercept 50 mg twice weekly (BIW; n = 379) or 50 mg once weekly (QW; n = 373) for 12 weeks by subcutaneous injection. All participants then received open-label etanercept 50 mg QW for 12 additional weeks, while remaining blinded to the randomization. A pharmacoeconomic questionnaire was administered at baseline, week 12 and week 24 of treatment. The questionnaire included employment status and changing job responsibilities and sick time taken due to psoriasis or PsA. The statistical methods included analysis of covariance, *t*-test, Fisher’s exact test and McNemar’s test. Last-observation-carried-forward imputation was used for missing data.

**Results:**

Employment was at least maintained from baseline to week 24 in both dose groups (56% [BIW/QW] and 60% [QW/QW] at baseline, 61% and 60%, respectively, at week 24). Among employed participants, the proportion of patients whose job responsibilities changed due to PsA decreased significantly from baseline to week 24 (17–23% to 5–8%; p < 0.01). Similar results were seen with job responsibility changes due to psoriasis (11–14% to 4%; p < 0.01). The number of monthly sick days also decreased from baseline to week 24 (2.4 days for both treatment groups to 0.7 (BIW/QW) and 1.1 (QW/QW); p ≤ 0.03 for each). No significant differences between the treatment groups were observed for any economic endpoint at any time point.

**Conclusions:**

For patients with moderate-to-severe plaque psoriasis and PsA, etanercept treatment resulted in reducing job responsibility changes due to disease and in reducing sick time. Effective treatment of psoriasis and PsA may reduce missed work days.

## Background

The prevalence of psoriatic arthritis (PsA) in patients with psoriasis is estimated to be as high as 30%, in contrast with a prevalence of <1% in the general population [1-3]. Both psoriasis and PsA negatively affect quality of life. Even patients with only mild psoriasis have reported problems in everyday life [4], including an inability to work [5,6]. PsA has been implicated in absences from work and career activities in previous studies [7,8]. However, more information is needed regarding employment, absenteeism and productivity in patients with PsA. A validated employment and productivity questionnaire that is specific for PsA is not currently available.

Etanercept, a fully human tumor necrosis factor-soluble receptor, is approved for the treatment of both PsA and moderate-to-severe plaque psoriasis based on demonstrated efficacy in treating both joint and skin symptoms.

In the PRESTA (Psoriasis Randomized Etanercept STudy in Patients with Psoriatic Arthritis) study [9-11], the efficacy, safety and patient-reported outcomes of two etanercept regimens were examined in patients with both moderate-to-severe psoriasis and PsA. Employment status and absenteeism were pre-defined outcomes of PRESTA and were determined via a pharmacoeconomic questionnaire.

The objective of this analysis is to examine the data from the pharmacoeconomic questionnaire to assess the impact of psoriasis plus PsA on working for pay and missed work days of patients who were treated with etanercept during the PRESTA study.

## Methods

**Figure 1:**
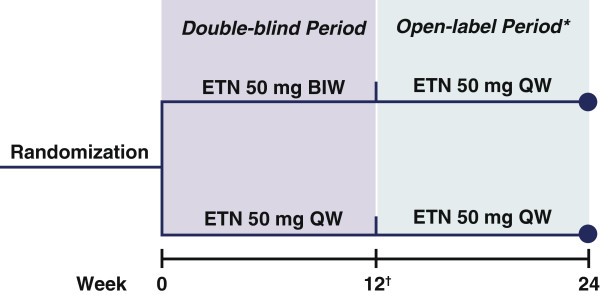
**Study design.** *Patients/physicians remained blinded to initial randomized treatment groups until end of open-label period. ^†^Primary endpoint was percent of patients achieving clear or almost clear status on PGA of psoriasis at week 12. BIW: twice weekly; ETN: etanercept; PGA: Physician Global Assessment; QW: once weekly.

The PRESTA study was conducted between December 2005 and March 2008, primarily in Europe and included sites in the Middle East, Asia-Pacific, Central America and South America [9]. Participants were enrolled from dermatology clinics based on having moderate/severe psoriasis and active PsA (with PsA evaluated by a rheumatologist or trained arthritis evaluator). In this multicenter, randomized study, patients received either etanercept 50 mg twice weekly (BIW) or 50 mg once weekly (QW) during the initial 12-week, double-blind period, followed by 50 mg QW during the subsequent 12-week, open-label period (initial randomization remained blinded; Figure 1).

Patients in the PRESTA study were aged at least 18 years, had clinically stable, moderate-to-severe plaque psoriasis with a body surface area involvement of ≥10% and had a Physician Global Assessment (PGA) of psoriasis ≥3 on a scale of 0 to 5 (0 = clear to 5 = severe). All patients had been diagnosed with PsA that included two or more swollen joints and two or more painful joints at screening and baseline, patient-reported joint pain for ≥3 months before screening and negative serum rheumatoid factor within 6 months of screening. Efficacy, safety and patient-reported outcomes for the PRESTA study have been published in detail [9-11].

### Pharmacoeconomic outcomes

A pharmacoeconomic questionnaire was used to assess the impact of PsA on employment, which included the following questions:

1. Are you currently employed (working for pay)?

2. Have you changed job responsibilities in the previous month because?

i. Your arthritis prevented you from continuing your old duties?

ii. Your psoriasis prevented you from continuing your old duties?

3. How many hours per week were you paid to work in the previous month?

4. How many sick days have you taken off from work in the previous month?

Patients were asked the same questions at baseline, week 12 and week 24. Sick leave was counted as number of days and was not adjusted for expected/planned number of working hours.

### Statistical analysis

All patients who completed the pharmacoeconomic questionnaire were included in the employment analyses. Only patients who were working for pay at baseline were included in the analyses of job changes and sick days. Statistical tests were two-tailed with an alpha of 0.05. Last-observation-carried-forward was used for imputation of missing values. Within-group changes from baseline in proportions were tested using McNemar’s test; changes from baseline for continuous endpoints were tested using t-tests. Between-group comparisons of proportions were tested using the Cochran-Mantel-Haenszel test; ANCOVA was used for between-group comparisons of continuous endpoints.

Stepwise logistic regression or linear regression analyses were conducted on predictors of employment outcomes at weeks 12 and 24. Separate models were run for those employed and not employed at baseline because follow-up questions about employment characteristics were only asked if the patient indicated that they were employed. The following parameters were included in models used for patients employed at baseline: age, gender, duration of psoriasis, duration of arthritis, treatment, baseline change of job responsibilities (yes or no) for psoriasis, baseline change of job responsibilities (yes or no) for arthritis, baseline sick days, baseline weekly hours worked. For those not in employment at baseline, only age, gender, duration of psoriasis, and duration of arthritis were included as the other information was not available.

The PRESTA study was designed and performed according to the guidelines for Good Clinical Practice and according to the version of the Declaration of Helsinki that was in place during the time the study was conducted.

The protocol and its amendments received independent ethics committee or institutional review board approval and regulatory review and approval before site initiation and recruitment of patients (Additional file 1). All participants signed and dated an approved informed consent form (ICF); the pharmacoeconomic questionnaire was referenced within the ICF.

## Results

A total of 752 patients were included in the modified intent-to-treat (mITT) population (n = 379, etanercept 50 mg BIW/QW; n = 373, etanercept 50 mg QW/QW). Baseline demographics and clinical characteristics for the overall mITT population have been described previously; no significant differences were observed between treatment groups, with the exceptions of prior methotrexate use and prior topical steroid use [9-11].

Most patients in this study who were of traditional working age (<65 years old) were employed for pay (61.0%). The proportion of male patients working for pay (68.1%) was higher than female patients (40.3%). Overall employment across the European, Latin American and Asian regions were similar (57.9%, 59.4% and 54.9%, respectively).

**Figure 2:**
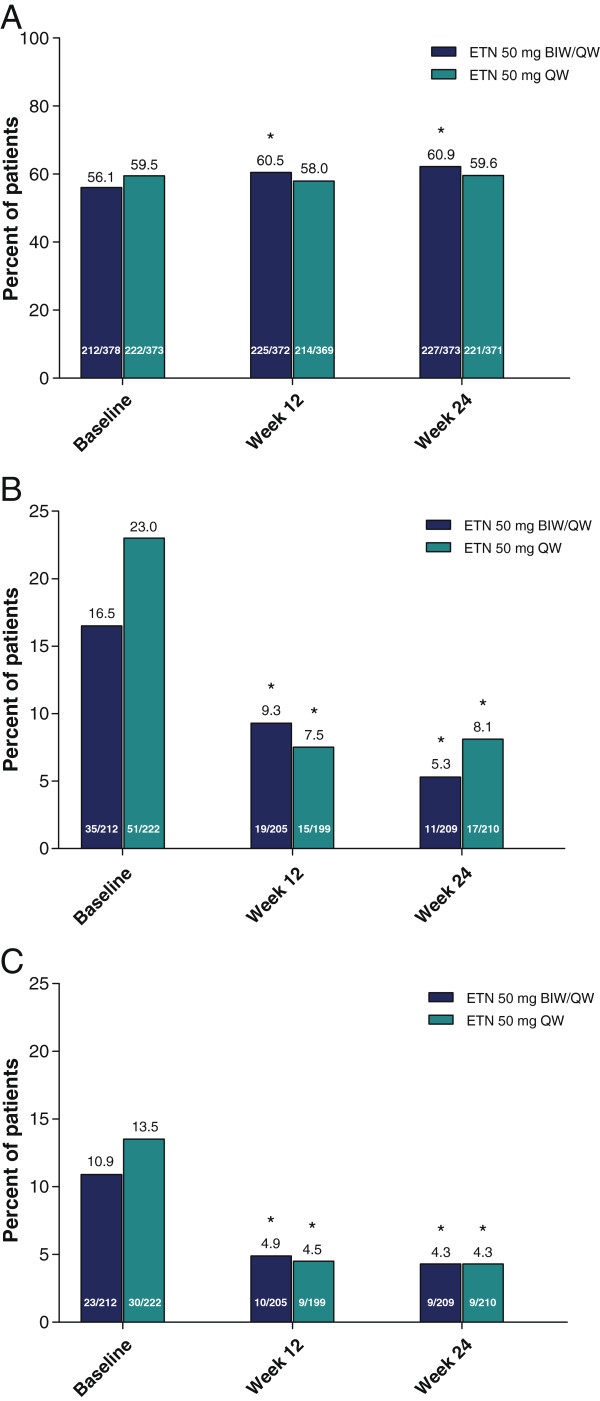
**Percent of patients employed and who changed job responsibilities due to arthritis symptoms or psoriasis symptoms. A**: Percent of patients employed. *p < 0.003 for within-group comparisons to baseline (McNemar’s test); p > 0.05 for between-group comparisons (Cochran-Mantel-Haenszel test). **B**: Percent of patients employed who changed job responsibilities in the previous month due to arthritis symptoms. *p < 0.004 for within-group comparisons to baseline (McNemar’s test); p > 0.05 for between-group comparisons (Cochran-Mantel-Haenszel test). **C**: Percent of patients employed who changed job responsibilities in the previous month due to psoriasis symptoms. *p < 0.009 for within-group comparisons to baseline (McNemar’s test); p > 0.05 for between-group comparisons (Cochran-Mantel-Haenszel test). BIW: twice weekly; ETN: etanercept; QW: once weekly.

**Figure 3:**
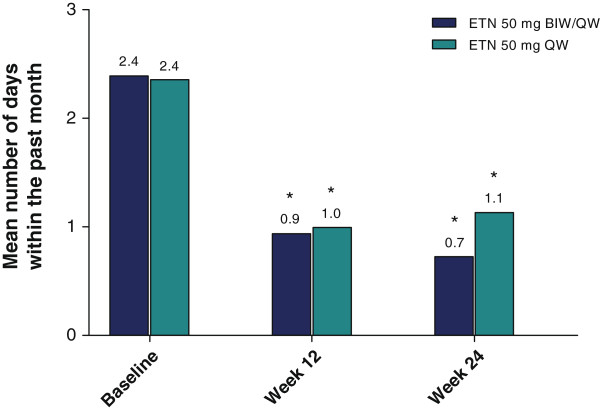
**Mean number of sick days taken during the past month.** *p < 0.001 for within-group comparisons to baseline in BIW/QW group (paired *t*-test); *p ≤ 0.031 for within-group comparisons to baseline in QW/QW group (paired *t*-test); p > 0.05 for between-group comparisons (ANCOVA). ANCOVA: analysis of covariance; BIW: twice weekly; ETN: etanercept; QW: once weekly.

Baseline demographics and disease characteristics differed significantly between patients who were employed and those who were not (Table 1). Those with more severe disease and poorer quality of life were less likely to be employed.The proportion of patients employed increased significantly in the 50 mg BIW/QW group from baseline (56%) to weeks 12 (60%) and 24 (61%; p ≤ 0.003), but not in the 50 mg QW/QW group (60%, 58% and 60%, respectively; Figure 2). However, differences between groups were not significant at any of the three time intervals, including baseline. The number of hours paid to work per week was 38.3 (BIW/QW group) and 39.2 (QW/QW group) at baseline, and remained steady at 39.5 and 41.8, respectively, by week 24 (p value was not significant between treatment groups).Significantly higher percentages of patients reported job changes due to arthritis (50 mg BIW/QW: 16.5%; 50 mg QW/QW: 23.0%) than due to psoriasis (10.9% and 13.5%, respectively, p < 0.0001 for pooled treatments) at baseline. The proportion of patients who reported having to change job responsibilities due to arthritis or psoriasis decreased significantly from baseline to weeks 12 and 24 in each group (p < 0.01; Figure 2). No significant differences were observed between groups at baseline or post-baseline in the proportion of patients who changed job responsibilities because of arthritis or psoriasis symptoms (p ≥ 0.336).At baseline, the mean number of monthly sick days taken was 2.4 in both treatment groups; these numbers declined from baseline to week 24 significantly in both groups (BIW/QW: 68.4%; QW/QW: 47.0%; p ≤ 0.031), without significant differences between the groups (p ≥ 0.268; Figure 3).

**Table 1:** Baseline demographics and clinical characteristics for employed vs. not employed patients (patient subpopulation <65 years old)

	**Employed (n = 431)**	**Not employed (n = 276)**	**Total (n = 707)**
Age, years	43.6 (9.4)*	47.4 (10.9)	45.1 (10.2)
Gender, male, n (%)	321 (74.5)^†^	133 (48.2)	454 (64.2)
Race, White, n (%)	386 (89.6)	238 (86.2)	624 (88.3)
Body mass index	27.7 (5.2)	28.3 (5.9)	28.0 (5.5)
Psoriasis disease duration, years	18.0 (10.2)	19.1 (12.6)	18.4 (11.2)
PsA disease duration, years	6.7 (6.7)	7.5 (7.5)	7.0 (7.0)
Physician Psoriasis Assessment (scale: 0–5; higher scores = worse psoriasis)	3.6 (0.6)*	3.7 (0.7)	3.6 (0.7)
PASI (scale: 0–72; higher scores = worse psoriasis)	18.5 (9.5)*	21.4 (11.1)	19.6 (10.3)
BSA affected, %	28.7 (21.4)*	34.7 (23.6)	31.0 (22.5)
PGA of arthritis (scale: 0–100; higher scores = worse arthritis)	48.9 (20.4)*	53.2 (21.5)	50.6 (20.9)
Swollen joints (0–68)	11.4 (13.9)*	14.5 (16.8)	12.6 (15.2)
Tender joints (0–72)	17.6 (16.8)*	22.0 (19.1)	19.4 (17.9)
DLQI, total (scale: 0–30; higher scores = worse dermatology-related quality of life)	11.6 (7.3)*	13.8 (7.7)	12.5 (7.3)
EQ-5D Utility (scale: 0–1; higher scores = better quality of life), mean	0.55 (0.28)*	0.38 (0.35)	0.48 (0.32)
EQ-5D VAS (scale: 1–100; higher scores = better health), mean	58.0 (20.7)*	51.9 (20.6)	55.6 (20.9)
HAQ DI (scale: 0–3; higher scores = more disability), mean	0.72 (0.59)*	1.20 (0.75)	0.91 (0.69)

*p < 0.01, for employed vs. not employed, one-way analysis of variance; ^†^p < 0.01, for employed vs. not employed, Fisher's Exact Test p-value (2-tail).

Mean (SD), unless otherwise noted.

In those patients who were employed at baseline, treatment with etanercept 50 mg BIW/QW remained a significant predictor of post-baseline employment following adjustment for predictive baseline characteristics (Table 2) (week 12: p = 0.001; week 24: p = 0.02). Treatment group was not a significant predictor of other post-baseline outcomes (hours worked, sick days, or post-baseline changes in employment due to arthritis or psoriasis), or of becoming employed in those patients not employed at baseline.

**Table 2:** Predictive ability of baseline characteristics on employment outcomes

**Independent variables (X variables used as predictors)**	**Dependent variables (Y variable being predicted)**							
	**Employed**		**Job responsibility change due to psoriasis**		**Job responsibility change due to PsA**		**Sick days**	
	**Week 12**	**Week 24**	**Week 12**	**Week 24**	**Week 12**	**Week 24**	**Week 12**	**Week 24**
Age	NS	NS	1.05 (1.00, 1.11)	NS	NS	1.04 (1.00, 1.09)	NS	NS
Female	NS	NS	3.1 (1.1, 9.0)	NS	NS	NS	NS	NS
Duration of psoriasis	NS	NS	NS	NS	NS	NS	NS	NS
Duration of PsA	NS	NS	NS	NS	NS	NS	NS	NS
ETN BIW/QW	5.2 (1.7, 15.7)	3.0 (1.1, 8.0)	NS	NS	NS	NS	NS	NS
Baseline job change due to psoriasis	NS	NS	22.9 (7.9, 66.3)	10.9 (4.0, 29.9)	3.1 (1.2, 8.6)	NS	2.6 (1.4, 3.8)	3.4 (1.8, 5.0)
Baseline job change due to PsA	NS	NS	NS	NS	4.9 (1.8, 12.9)	6.3 (2.7, 14.6)	NS	-1.7 (-3.0, -0.4)
Baseline sick days*	NS	0.94 (0.90, 0.99)	NS	NS	NS	NS	0.17 (0.11, 0.24)	0.15 (0.08, 0.22)
Baseline hours worked^†^	1.05 (1.02, 1.08)	NS	NS	0.96 (0.93, 0.99)	0.96 (0.94, 0.98)	0.95 (0.92, 0.97)	-0.04 (-0.06, -0.01)	-0.04 (-0.07, -0.01)

NS = Not significant.

Data for all dependent variables are odds ratio (95% confidence intervals) except Sick days which show slope (95% confidence intervals). *Odds are for a one-day increase in baseline sick days. ^†^Odds are for a one-hour increase in baseline hours worked.

In patients employed at baseline, the probability of continued employment was also positively associated at week 12 with the number of hours worked at baseline (p < 0.0001) and negatively associated at week 24 with the number of sick days at baseline (p < 0.001). Female gender (p = 0.03) and fewer weekly hours worked (p = 0.02) at baseline were predictors of changing job responsibilities due to psoriasis at week 12 and week 24, respectively; neither was predictive of changing job responsibilities due to PsA at either post-baseline observation. The probability of changing job responsibilities due to PsA was negatively associated with baseline hours worked at week 12 (p = 0.005) and week 24 (p < 0.001) and also positively associated with older age at week 24 (p < 0.001). Baseline sick days and fewer weekly hours worked at baseline were predictive of more sick days recorded at week 12 and week 24 (all p < 0.01). In patients not employed at baseline, the probability of becoming employed at weeks 12 and 24 was lower for older patients and females. Duration of psoriasis and duration of psoriatic arthritis were not predictive of employment, changing job responsibilities or sick days at either week 12 or 24.

## Discussion

In the PRESTA trial, treatment of psoriasis and PsA patients with etanercept over 24 weeks resulted in maintenance of employment in both treatment groups. Patients in the higher dose group had statistically significant improvements in the proportions employed, but the baseline proportions of employed patients were lower than in the 50 mg QW/QW dose group. In addition to the percentage of patients remaining employed, the number of hours worked each week improved slightly but not significantly. The majority of senior patients (aged ≥65 years) were not employed, suggesting they were retired and not seeking work. Among the working age patients (aged <65 years) the group who were not employed had more severe disease relative to the employed group. The percentage of employed patients from Europe who were <65 years old in this study (61.2%, 95% CI: 57.2% – 65.1%) is slightly less than the percentage employed in the overall population in the European area who were aged 15 to 64 at the end of 2006 (65%) and 2007 (66%) [12].

One of the strengths of this study is that the pharmacoeconomic questionnaire could distinguish between employment changes due to skin disease symptoms versus arthritis symptoms. In both treatment groups, a larger proportion of patients reported changing job responsibilities due to arthritis symptoms than due to psoriasis symptoms. The proportion of patients who had to change jobs for either reason decreased significantly from baseline to the end of the study.

There was a >47% reduction in sick days after 12 weeks of treatment. These data are comparable with a previous study that looked at treatment of active PsA with etanercept over 24 months [13]. Unlike the current study, those investigators observed a nonsignificant decline in missed work days (absenteeism for any reason) from 0.7 days per 2 weeks at baseline to 0.3 days per month after 2 years of treatment (p = 0.3436).

Previous studies have shown that patients with PsA have an elevated rate of work disability relative to normal populations, consistent with our findings at baseline [14]. In addition, etanercept treatment of PsA has been seen to result in impediments to labor (paid or unpaid) declining significantly over 2 years (p < 0.001) [13]. Our study did not look specifically at impediments to work, but the data indirectly support the previous research.

### Limitations

The PRESTA study was not designed to specifically study employment. Furthermore, the pharmacoeconomic questionnaire used in the PRESTA study has not been validated in other clinical trials. At the time this study was conducted, work productivity questionnaires, including the Work Productivity and Activity Index for psoriasis [15] and for rheumatoid arthritis [16] were not widely used. Although the pharmacoeconomic questionnaire has not undergone extensive validation, the results are similar to other work productivity instruments [13,14]. The pharmacoeconomic questionnaire can also be viewed as having face validity – the quality of prompting patients to provide the appropriate information. Both arms of PRESTA involved treatment with etanercept, thus comparisons with placebo were not available. However, one might expect employment to decline slowly for patients with PsA given the cumulative damage to joints that can occur. The lack of a placebo arm in PRESTA raises the possibility that some of the improvement observed with etanercept treatment could be due to regression to the mean although the lack of association between disease duration and employment outcomes reduces the likelihood that this problem was the primary determinant of these results.

## Conclusions

For patients with moderate to severe psoriasis and PsA, etanercept provides economic value, in part, by reducing changes in job responsibilities and missed work days.

## Abbreviations

BIW: Twice weekly; DLQI: Dermatology Life Quality Index; EQ-5D Utility: EuroQol – 5 Dimension Utility index; EQ-5D VAS: EuroQol – 5 Dimension visual analog scale; HAQ DI: Health Assessment Questionnaire Disability Index; mITT: Modified intent-to-treat; PASI: Psoriasis Area and Severity Index; PsA: Psoriatic arthritis; QW: Once weekly; SD: Standard deviation.

## Competing interests

RLB, TW, LM, and RP were employees of Pfizer Inc during the PRESTA study and subsequent analyses. RG has been a paid lecturer and investigator for Pfizer, MSD, Abbvie, Janssen, Celgene and Actelion, and is a member of the Danish National Advisory Boards for MSD, Janssen and Abbvie. SK has been a principal investigator for Abbvie, Janssen, Actelion, Pfizer and a National Advisory Board member for Janssen. WL was an employee of Quintiles Inc. and a paid contractor to Pfizer Inc during the PRESTA study and development of this manuscript.

## Authors’ contributions

RLB and TW were involved in the design and implementation of the PRESTA study and provided data interpretation. RG and SK were investigators in the PRESTA study and provided data interpretation. WL and RP provided statistical analyses. LM provided valuable clinical insight and data interpretation. All authors provided reviews of each draft of the manuscript and approved the final draft for submission.

## Pre-publication history

The pre-publication history for this paper can be accessed here:

http://www.biomedcentral.com/1471-5945/14/14/prepub

## Supplementary Material

Additional file 1 List of independent ethics committee and institutional review boards for the PRESTA study. Click here for file

## References

[B1] Gladman DD Psoriatic arthritis Dermatol Ther 2004 17 350 363 10.1111/j.1396-0296.2004.04038.x 15379770

[B2] Gottlieb AB Chao C Dann F Psoriasis comorbidities J Dermatol Treat 2008 19 5 21 10.1080/09546630701364768 18273720

[B3] Mease P Management of psoriatic arthritis: the therapeutic interface between rheumatology and dermatology Curr Rheumatol Rep 2006 8 348 354 10.1007/s11926-006-0064-9 16973108

[B4] Stern RS Nijsten T Feldman SR Margolis DJ Rolstad T Psoriasis is common, carries a substantial burden even when not extensive, and is associated with widespread treatment dissatisfaction J Investig Dermatol Symp Proc 2004 9 136 139 10.1046/j.1087-0024.2003.09102.x 15083780

[B5] Dubertret L Mrowietz U Ranki A van de Kerkhof PC Chimenti S Lotti T Schäfer G European patient perspectives on the impact of psoriasis: the EUROPSO patient membership survey Br J Dermatol 2006 155 729 736 10.1111/j.1365-2133.2006.07405.x 16965422

[B6] Gelfand JM Feldman SR Stern RS Thomas J Rolstad T Margolis DJ Determinants of quality of life in patients with psoriasis: a study from the US population J Am Acad Dermatol 2004 51 704 708 10.1016/j.jaad.2004.04.014 15523347

[B7] Zachariae H Zachariae R Blomqvist K Davidsson S Molin L Mørk C Sigurgeirsson B Quality of life and prevalence of arthritis reported by 5,795 members of the Nordic Psoriasis Associations. Data from the Nordic Quality of Life Study Acta Derm Venereol 2002 82 108 113 10.1080/00015550252948130 12125937

[B8] Radtke MA Reich K Blome C Rustenbach S Augustin M Prevalence and clinical features of psoriatic arthritis and joint complaints in 2009 patients with psoriasis: results of a German national survey J Eur Acad Dermatol Venereol 2009 23 683 691 10.1111/j.1468-3083.2009.03159.x 19309433

[B9] Sterry W Ortonne JP Kirkham B Brocq O Robertson D Pedersen RD Estojak J Molta CT Freundlich B Comparison of two etanercept regimens for treatment of psoriasis and psoriatic arthritis: PRESTA randomised double blind multicentre trial BMJ 2010 340 c147 10.1136/bmj.c147 20124563

[B10] Gniadecki R Robertson D Molta CT Freundlich B Pedersen R Li W Boggs R Zbrozek AS Self-reported health outcomes in patients with psoriasis and psoriatic arthritis randomized to two etanercept regimens J Eur Acad Dermatol Venereol 2012 26 1436 1443 10.1111/j.1468-3083.2011.04308.x 22035157

[B11] Prinz JC Fitzgerald O Boggs RI Foehl J Robertson D Pedersen R Molta CT Freundlich B Combination of skin, joint and quality of life outcomes with etanercept in psoriasis and psoriatic arthritis in the PRESTA trial J Eur Acad Dermatol Venereol 2011 25 559 564 10.1111/j.1468-3083.2010.03838.x 20840349

[B12] Short-Term Labour Market Statistics: Employment Rate http://stats.oecd.org/Index.aspx?QueryId=38900

[B13] Gladman DD Bombardier C Thorne C Haraoui B Khraishi M Rahman P Bensen W Syrotuik J Poulin-Costello M Effectiveness and safety of etanercept in patients with psoriatic arthritis in a Canadian clinical practice setting: the REPArE trial J Rheumatol 2011 38 1355 1362 10.3899/jrheum.100698 21572156

[B14] Wallenius M Skomsvoll JF Koldingsnes W Rødevand E Mikkelsen K Kaufmann C Kvien TK Work disability and health-related quality of life in males and females with psoriatic arthritis Ann Rheum Dis 2009 68 685 689 10.1136/ard.2008.092049 18511544

[B15] Pearce DJ Singh S Balkrishnan R Kulkarni A Fleischer AB Feldman SR The negative impact of psoriasis on the workplace J Dermatol Treat 2006 17 24 28 10.1080/09546630500482886 16467020

[B16] Zhang W Bansback N Boonen A Young A Singh A Anis AH Validity of the work productivity and activity impairment questionnaire–general health version in patients with rheumatoid arthritis Arthritis Res Ther 2010 12 R177 10.1186/ar3141 20860837 PMC2991008

