# Supplementary material for: Employment is maintained and sick days decreased in psoriasis/psoriatic arthritis patients with etanercept treatment
Source: BMC Dermatol. 2014 Aug 5;14:14. doi: 10.1186/1471-5945-14-14 (PMC4147874; doi:10.1186/1471-5945-14-14)
Supplement: Additional file 1 — List of independent ethics committee and institutional review boards for the PRESTA study. [file 1471-5945-14-14-S1.docx]

**Additional file 1:** List of independent ethics committee and institutional review boards for the PRESTA study

- University of Debrecen Regional and Institutional Research Ethics Committee of the Medical and Health Science Center (DEOEC), Debrecen, Hungary
- Regional and Institutional Committee of Science and Research Ethics, Budapest, Hungary
- Comitato Etico Istituto Dermopatico Dell'Immacolata, Rome, Italy
- Egeszsegiigyi Tudomainyos Tanaics, Klinikai Farmakologiai Etikai Bizottsaga, Budapest, Hungary
- Szegedi Tudomanyegyetem Szent-Gyorgyi Albert Orvos es Gyogyszeresztudomanyi Centrum IKEB, Szeged, Hungary
- Pecsi Tudomanyegyetem Szent-Gylogyi Albert Orvos es Egeszsegtudomanyi Centrum, IKEB, Pecs, Hungary
- Miskolc County Semmelweis Hospital University Teaching Hospital Local Ethic Committee, Miskolc, Hungary
- Jasz-Nagykun-Szolnok Megyei Hetenyi Geza Korhaz-Rendelointezet Intezeti Kutatasetikai Bizottsag, Debrecen, Hungary
- USL 8 Ethical Committee, Selargius, Italy
- Spedali Civili –Berscia Azienda Ospedaliera Ethics Committee, Brescia, Italy
- Ethical Committee University Hospital Authority Vittorio Emanuele-Ferrarrotto-S. Bambino , Catania, Italy
- Segreteria Scientifica del Comitato Etico, Milan, Italy
- Ethics Committee Azienda Policlinico Umberto I Universito degli Studi di Romo "la Sapienza", Rome Italy
- Comitato Etico Locale per la Sperimentazione Clinica dei Medicinali dell'AOUS, Sienna, Italy
- Comitato di Etica Universita degli Studi di Parma, Parma, Italy
- National Ethics Committee, Athens, Greece
- Hacettepe University Medical Faculty Medical, Surgical And Drug Research Ethics Committee, Sihhiye, Turkey
- Ethik-Kommission des Landes Federal State of Berlin, Berlin, Germany
- Landesamt fiir Gesundheit und Soziales Berlin Postfach Berlin, Germany
- Ethik-Kommission der Medizinischen Fakultat der Ludwig-Maximilians-Universitat Munich, Germany
- Ethik-Kommission der Arztekammer Hamburg, Hamburg Germany
- Ethik-Kommission an der Medizinischen Fakultat der Rheinischen Friedrich-Wilhelms-Universitat, Bonn Institut fur Pharmakologie und Toxikologie Reuterstrate, Bonn, Germany
- Ethik-Kommission der LandesUrztekammer Brandenburg Dreifcrtstrafe, Cottbus Germany
- Ethik-Kommission der Friedrich-Schiller-Universitilt, Jena, Germany
- Landesamt fiir Gesundheit und Soziales, Ethik-Kommission des Landes Berlin, Berlin, Germany
- Ethik-Kommission der Medizinischen Fakultaet Universitaet Rostock Institut fuer Rechtsmedizin, Rostock, Germany
- Ethikkommission der Med. Fakultiit der Universitiit Erlangen­Niimberg Universitiitsstr, Erlangen, Germany
- Ethik-Kommission der Med. Fakultat der Universitat Wiirzburg, Wiirzburg Germany
- Ethik-Kommission des Fachbereichs Medizin Der Johann-Wolfgang-Universitaet Frankfurt, Frankfurt am Main, Germany
- Ethikkommission des Landes Berlin Landesamt fiir Gesundheit und Soziales Berlin, Berlin, Germany
- Ethik-Kommission bei der Arztekammer Niedersachsen Unterkommisison zur Beurteilung medizinischer Forschung am Menschen, Hannover Germany
- Regionale METC Zuidwest Holland, The Netherlands
- METC Zuidwest Holland, Voorburg, The Netherlands
- CHU Sart Tilman Commission d'Ethique, Insitut de Pathologie, Liege, Belgium
- Uniwersytet Mikolaja Kopemika w Toiuniu Collegium Medicum im L. Rydygiera w Bydg6szczy Komisja Bioetyczna, Bydgoszcz, Poland
- CCPPRB (Haute-Normandie), Hopital Charles Nicolle, Rouen, France

- De Videnskabetiske Komiteer for Kepenhavns og Frederiksberg Kommuner Kobenhavns Kommune, Copenhagen, Denmark
- Sub-Committee on Medicinal Research Ethics, Tukija Ministry of Social Affairs and Health, Helsinki, Finland
- Regional Ethics Committee in Uppsala, Regionala etikprovningsniimnden, Uppsala, Sweden
- Liikemedelsverket Fannakoterapeutiska enheten, Uppsala, Sweden
- St. Vincent's Health Human Research Ethics Committee, St. Vincent's Hospital, Melbourne, Australia
- Human Research Ethics Committee (Western Zone), Liverpool, NSW, Australia

- National Taiwan University, Taipei, Taiwan
- Chung Gung Memorial Hospita, Kaohsiung County, Taiwan
- Consejeria de Salud Secretaria General de Calidad y Eficiencia Comite, Autonomico de Ensayos Clinicos Avd. Innovacion s/n. Edificio Arena, Seville, Spain
- Hospital Ramon y Cajal CEIC, Madrid, Spain
- Hospital Santa Creu I Sant Pau CEIC, Barcelona, Spain
- Registro General del Complejo Hospitalario Materna Insular, Las Palmas, Gran Canaria, Spain
- Secretaria del CEIC-IMAS Institut Municipal d'Investigacio Medica (IMIM), Barcelona, Spain
- CEIC Locales Secretaria Administrativa del CEIC-E, Direccion de Farmacia Departamento de Sanidad, Vitoria-Gasteiz, Spain
- Hospital Donostia, San Sebastian, Spain
- Comissao de Etica para a investigacao Clf nica (CEIC) Parque da Saude de Lisboa, Lisbon, Portugal
- Gabinete de Apoio a lnvestigacao Edificio S Jeronimo Hospitais da Universidade de Coimbra, Coimbra, Portugal
- Ethikkommission der Stadt Wien, Magistrat der Stadt Wien, Vienna, Austria
- Ethikkommission der Medizinischen Universitat, Graz LKH-Upiversitatsklinikum, Graz, Austria
- Institutional Review Board, Seoul National University Hospital, Seoul, Korea
- Institutional Review Board, Asan Medical Center, Seoul, Korea
- Cantonal Ethics Committee, SPUK filr Spezialfacher Universitats Spital, Zurich, Switzerland
- Faculte de Biologie et de Medecine Commission d'Ethique de la Recherche Clinique, Universite de Lausanne, Switzerland

- Kingdom of Saudia Arabia King Faisal Specialist Hospital and Research Centre, Riyadh, Saudi Arabia
- CEPI Investigation Protocols Ethics Committee, Medicine Faculty of the Buenos Aires University, Buenos Aires, Argentina
- Comite De Docencia E Investigacion " y Comite de Bioetica del Moal Gral. De Agodos "Dr. Cosme Argerich", Buenos Aires, Argentina
- Teaching and Research Committee from Hospital Nacional Profesor Alejandro Posadas El Palomar, Moron, Buenos Aires, Argentina
- CEMIC Centro de Educacion Medica E Investigaciones Clinicas ‘Norberto Quirno’, Buenos Aires, Argentina
- Unidad de Investigacion en Enfermedades Cronico-Degenerativas, SC Bioethics Committee, Guadalajara, Mexico
- CBIC Clinical Investigation Bioethics Committee, Celia Ovadio Durango, Mexico
- St. Thomas Ethics Committee, St. Thomas Hospital, London, UK
- Royal Free Hospital Local Research Ethics Committee, London, UK
- South West MREC, Dartington, UK
- Alsford & Trafford Research Ethics Committee, Manchester, UK
- South East Wales LREC, Cardiff, UK
- Grampian LREC, Grampian NHS Board, Aberdeen, UK
- Ethics Committee, Institute of Rheumatology, Resavska, Beograd, Serbia
- Institute of Rheumatology-Niska Banja Ethics Committee, Niska Banja, Serbia
- Ethical Committee of Clinical Center-Zemun, Vukova, Zemun, Serbia
- Ethics Committee, Clinical Center - Vojvodina Hajduk Veljkova, Novi Sad, Serbia
- Ethics Commitee for Multicentric Trials Teaching Hospital, Motol V Ovalu, Prague , Czech Republic
- Ethics Committee at Rheumatological Institute, Na Slupi, Prague, Czech Republic
- Comite de Etica de Riesgo de Fractura, Bogota, Colombia
